# An Improved Synthesis Phase Unwrapping Method Based on Three-Frequency Heterodyne

**DOI:** 10.3390/s22239388

**Published:** 2022-12-01

**Authors:** Jiangtao Liu, Peng Tian, Hongru Li, Hao Wei, Guoliang Deng, Shouhuan Zhou, Zeyu Ma, Wenwu Wang, Liang He

**Affiliations:** 1 College of Electronics and Information Engineering, Sichuan University, Chengdu 610065, China; 2 School of Mechanical Engineering, Sichuan University, Chengdu 610065, China

**Keywords:** 3D measurement, fringe projection profilometry, phase unwrapping, synthesis phase, three-frequency heterodyne

## Abstract

An improved three-frequency heterodyne synthesis phase unwrapping method is proposed to improve the measurement accuracy through phase difference and phase sum operations. This method can reduce the effect of noise and increase the equivalent phase frequency. According to the distribution found in the phase difference calculation process, the Otsu segmentation is introduced to judge the phase threshold. The equivalent frequency obtained from the phase sum is more than those of all projected fringe patterns. In addition, the appropriate period combinations are also studied. The simulations and related experiments demonstrate the feasibility of the proposed method and the ability to improve the accuracy of the measurement results further.

## 1. Introduction

Fringe projection profilometry (FPP) is an optical three-dimensional (3D) profile measurement technique [1,2,3,4], which is a non-contact active measurement method to obtain the 3D profile of an object by projecting and collecting fringe patterns. With the development of digital projectors and sensor devices, this method achieves high accuracy at a low cost. Therefore, this method has many applications in reverse engineering, mechanical assembly, biomedicine, heritage conservation, and other fields [5,6,7].

The core content in structured light 3D measurement is obtaining the continuous phase of the object [8,9]. The phase acquisition is divided into Fourier transform profilometry (FTP) [10] and phase-shifting profilometry (PSP) [11]. For FTP, the fundamental frequency component is filtered out in the frequency domain after the collected image is transformed by the Fourier transform. Then, the Fourier inverse transform is used to obtain the phase. This method only needs one fringe image [12,13] and has good performance in high-speed measurement, but the measurement accuracy of this method is low. However, according to the principle of PSP, the phase is obtained by projecting multiple groups of phase-shifting fringe patterns to the object and performing the point-to-point operations on the collected fringes. This method effectively avoids the influence between adjacent points and has high measurement accuracy [14]. Since these methods extract the phase by tangent calculation, the phase values range from −π to π. Therefore, a phase unwrapping process is required to obtain a continuous phase.

Among the phase unwrapping methods, they can be broadly classified into two categories, spatial phase unwrapping (SPU) [15] and temporal phase unwrapping (TPU) [16]. The SPU [17,18,19] algorithms unwrap the phase by the phase value of adjacent pixels. However, one problem with this SPU algorithm is that the phase errors can spread to other locations. In contrast, the TPU algorithm does not have this problem [20,21,22,23]. The basic idea of this algorithm is to make the frequency of fringes change with time, and the fringe patterns are regarded as a sequence of the time axis. Many groups of fringes are projected during the measurement process, and TPU methods improve the signal-to-noise ratio (SNR) of phase change. Among the TPU algorithms, many researchers used the multi-frequency heterodyne (MFH) algorithm because of its high measurement accuracy. The MFH algorithm can be divided into the dual-frequency heterodyne (DFH) [24,25], the three-frequency heterodyne (TFH) [26,27], and also can be extended to more frequencies [16,28,29]. Compared with the DFH and more frequency methods, the TFH phase unwrapping algorithm has a higher resolution of details, a larger measurement field, and a higher measurement accuracy. Additionally, the required data volume is moderate. Therefore, the TFH algorithm is selected for phase unwrapping in this study.

The phase difference and phase sum were first proposed in the 1970s [30,31,32,33], and, after this, many scholars have studied this technique [34,35,36]. The traditional TFH phase unwrapping algorithm obtains a synthetic phase covering the whole measurement field by phase difference operation. The synthetic phase calculates the order of the minimum period phase, then the continuous phase is obtained. The increase in noise and the decrease in equivalent frequency will lead to phase jumps and a reduction in measurement accuracy. An improved TFH synthesis phase unwrapping method is proposed to solve these problems. The phase sum operation is introduced into the TFH algorithm, and the use of the phase sum operation can obtain a higher frequency phase, which can achieve a higher sensitivity gain and SNR [34,35,36,37]. The method improves the phase sum and phase difference operations to obtain better measurement results. The Otsu segmentation algorithm [38] calculates the threshold according to the statistics distribution in the phase difference operation process. The phase sum operation is introduced into the TFH algorithm, and the use of the phase sum operation can obtain a higher frequency phase. The frequency of the phase sum is more than those of all the projected fringes, while the range that can be measured is larger after the first and second phases’ synthesis operations. The relationship between fringe period combinations and corresponding measurement accuracy is also studied to help select optimal fringes. Simulations and experiments prove that this method can improve accuracy and decrease errors caused by noise.

## 2. Principle

**Figure 1:**
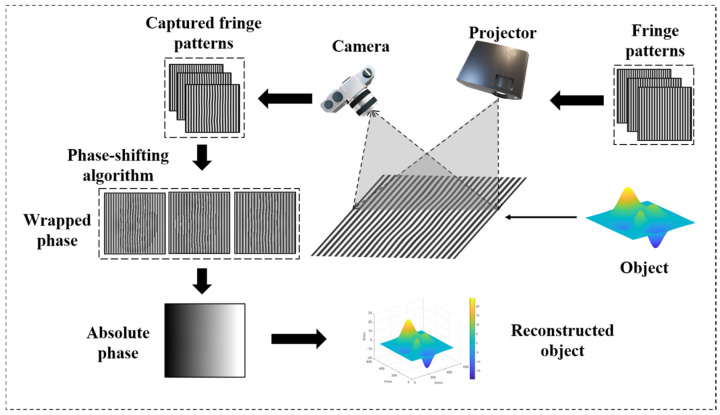
Schematic diagram of an FPP setup.

The FPP measurement system comprises a projector, a camera, a processing unit (computer), and a working plane. In a 3D measurement process, a computer generates pre-designed sinusoidal fringe patterns, which are then projected onto the object’s surface by a projector. The camera captures the deformed fringes modulated by the object, then uses the phase extraction formula to obtain the wrapped phase. The schematic diagram is shown in Figure 1.

### 2.1. Wrapped Phase Extraction

In *N*-step PSP, the projected fringe patterns $I_{n}^{p}$ (n=1,…,N; N≥3) can be denoted as

(1) $$I_{n}^{p}\left(x^{p},y^{p}\right)=a^{p}+b^{p}\cos\left[2\pi fx^{p}-2\pi n/N\right],$$

where $\left(x^{p},y^{p}\right)$ is the pixel coordinate of the projector, $a^{p}$ is the average intensity, $b^{p}$ is the amplitude, f is the frequency of the projected fringe ($f=\frac{1}{T}$, T is the period of the projected fringe), n represents the phase-shifting index, and N is the phase-shifting step number [14].

The intensity of deformed fringe patterns captured by a camera can be expressed as

(2) $$I_{n}\left(x,y\right)=A\left(x,y\right)+B\left(x,y\right)\cos\left[\varphi \left(x,y\right)-2\pi n/N\right],$$

where (x,y) is the pixel coordinate of the camera, A(x,y) is the average intensity relating to the pattern brightness and background illumination, B(x,y) is the intensity modulation relating to the pattern contrast and surface reflectivity, and φ(x,y) is the corresponding wrapped phase which can be extracted by [14]

(3) $$\varphi \left(x,y\right)=\tan^{-1}\frac{\sum_{n=1}^{N}I_{n}\left(x,y\right)\sin\left(2\pi n/N\right)}{\sum_{n=1}^{N}I_{n}\left(x,y\right)\cos\left(2\pi n/N\right)}.$$

It can be noted that there are three unknown quantities, A(x,y), B(x,y), and φ(x,y), in Equation (2), so at least three fringe patterns are needed to calculate the wrapped phase φ(x,y). Due to the different projected fringe frequencies used in the TFH method, the wrapped phase of each frequency needs to be obtained for further unwrapping.

### 2.2. Principle of Phase Unwrapping

The phase values calculated by Equation (3) range from −π to π and they will have a phase jump of 2π. To obtain a continuous phase, the wrapped phase plus integer multiple of 2π is needed, which is given by

(4) Φ(x,y)=φ(x,y)+2πk(x,y),

where φ(x,y) is the wrapped phase, Φ(x,y) is the unwrapped phase, and k(x,y) is the fringe order. The main task of phase unwrapping is to calculate the correct fringe orders k(x,y).

### 2.3. The TFH Phase Unwrapping Algorithm

#### 2.3.1. The Traditional TFH Phase Unwrapping Algorithm

In the traditional TFH phase unwrapping algorithm, three groups of fringes with frequencies of $f_{1}$, $f_{2}$, and $f_{3}$ ($f_{1}>f_{2}>f_{3},\;f_{i}=\frac{1}{T_{i}},\;i=1,2,3$) are projected onto the target object. Then, the wrapped phase $\varphi_{1}$, $\varphi_{2}$, and $\varphi_{3}$ are obtained by Equation (3). The synthetic phases $\varphi_{12d}$, $\varphi_{23d}$, and $\varphi_{123d}$ can be expressed, respectively, as

(5) $$\begin{array}{c} \{\begin{array}{c} \varphi_{12d}=\{\begin{array}{c} \varphi_{1}-\varphi_{2},\;\varphi_{1}\geq \varphi_{2}\\ \varphi_{1}-\varphi_{2}+2\pi ,\;\varphi_{1}<\varphi_{2} \end{array}\\ \varphi_{23d}=\{\begin{array}{c} \varphi_{2}-\varphi_{3},\;\varphi_{2}\geq \varphi_{3}\\ \varphi_{2}-\varphi_{3}+2\pi ,\;\varphi_{2}<\varphi_{3} \end{array} \end{array}\\ \varphi_{123d}=\{\begin{array}{c} \varphi_{12d}-\varphi_{23d},\;\varphi_{12d}\geq \varphi_{23d}\\ \varphi_{12d}-\varphi_{23d}+2\pi ,\;\varphi_{12d}<\varphi_{23d} \end{array} \end{array}$$

where subscript d refers to phase difference operation. The equivalent frequencies of synthetic phases meet $f_{12d}=f_{1}-f_{2}$, $f_{23d}=f_{2}-f_{3}$, and $f_{123d}=f_{12d}-f_{23d}$ and $T_{12d}=1/f_{12d}$, $T_{23d}=1/f_{23d}$, and $T_{123d}=1/f_{123d}$.

The obtained synthetic phase is equivalent to the extract phase from equivalent frequency fringes. The equivalent phase with a larger period is obtained by phase difference operation. If the fringe period is chosen appropriately, the synthetic phase $\varphi_{123}$ with a period covering the whole measurement field can be obtained after two phase difference operations. $\Phi \left(x,y\right)=\varphi_{123d}\left(x,y\right)$ is the continuous phase that helps calculate the fringe orders.

The fringe order k of $\varphi_{h}$ and continuous phase $\Phi_{h}$ can be obtained by Equation (6) as

(6) $$\begin{array}{c} k=round\left[\frac{\Phi_{l}\left(f_{h}/f_{l}\right)-\varphi_{h}}{2\pi }\right]\\ \Phi_{h}=\varphi_{h}+2\pi k \end{array}$$

where round[⋅] is the symbol indicating the nearest integer to the value. $f_{l}$ and $f_{h}$ are the frequencies of low-frequency and high-frequency fringes, $\varphi_{h}$ is the high-frequency wrapped phase, and $\Phi_{l}$ and $\Phi_{h}$ are the low-frequency and high-frequency continuous phases [29]. By repeatedly applying the process of recovering the high-frequency phase with the low-frequency phase, the final continuous phase with the highest frequency is obtained.

For the traditional TFH phase unwrapping algorithm, it is easy to misjudge the phase when the phase values are closer due to the noise effect in the phase difference calculation process, which will lead to the phase jump with the value of 2π. In addition, the reduction of the equivalent frequency will also affect the measurement accuracy.

#### 2.3.2. The Proposed Algorithm

**Figure 2:**
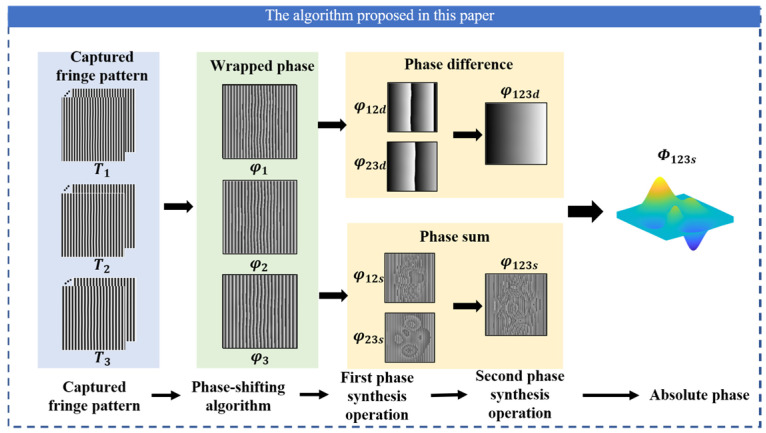
Schematic diagram of the proposed algorithm.

Figure 2 shows the schematic diagram of the proposed algorithm. Both phase difference and phase sum operations are introduced to obtain synthetic phases, which could conduct the final continuous phase. Firstly, three groups of fringes with different periods are projected, and the periods meet $T_{1}<T_{2}<T_{3}$. Similar to the traditional method, by calculating the phase difference with Equation (5), the phase differences $\varphi_{12}{}_{d}$ and $\varphi_{23}{}_{d}$ can be obtained.

**Figure 3:**
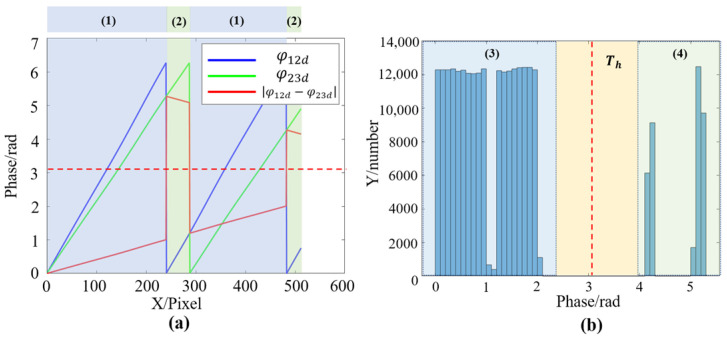
Phase difference calculation results. (**a**) Synthetic phases and the absolute values of their difference. (**b**) Histogram graph of the $\left|\varphi_{12d}-\varphi_{23d}\right|$, the red dotted line is the possible threshold $T_{h}$.

After this first phase difference calculation, the phase with the larger equivalent period is achieved. However, during the second phase difference calculation for $\varphi_{123d}$, the positions with very small phase differences are sensitive to noise. The possible noise will make the phase fluctuate, which is easy to cause misjudgment, and eventually lead to phase jumps. To investigate how to obtain robust phase difference results in this process, the lateral change of the two synthetic phases was observed after the first phase difference calculation, and it was found that the phase difference absolute value $\left|\varphi_{12d}-\varphi_{23d}\right|$ is mainly divided into two parts, (1) and (2), respectively, as shown in Figure 3a.

It can be observed from Figure 3a that the distribution of phase difference in (1) and (2) is discontinuous, and the value in the (1) region is smaller than that in the (2) region. There is a steep jump in the change of phase difference value. In addition, the statistics of phase differences are shown in Figure 3b. It also can be found that the difference is mainly divided into two parts, (3) and (4). Part (3) corresponds to the region (1), which has smaller values, and part (4) corresponds to the region (2), which has larger values. It can be clearly noticed that there is a gap between part (3) and part (4) from Figure 3b.

Based on the above findings, the threshold can be set to carry out the correct phase difference operation in the second phase difference calculation process. This algorithm uses the Otsu thresholding segmentation algorithm to calculate the intermediate thresholds of part (3) and part (4). The Otsu method is an adaptive threshold segmentation algorithm, and it can quickly calculate the interclass data threshold by the maximum variance between two data classes after segmentation. Therefore, the threshold $T_{h}$ can be obtained by

(7) $$T_{h}=OTSU\left(\left|\varphi_{12d}-\varphi_{23d}\right|\right),$$

where OTSU(⋅) is the Otsu operation. By comparing the phase difference absolute value and the threshold value, the improved second phase difference calculation can be expressed as

(8) $$\begin{array}{c} \varphi_{12}{}_{3d}=\{\begin{array}{c} \varphi_{12d}-\varphi_{23d},\;\left|\varphi_{12d}-\varphi_{23d}\right|\leq T_{h}\\ \varphi_{12d}-\varphi_{23d}+2\pi ,\;\left|\varphi_{12d}-\varphi_{23d}\right|>T_{h} \end{array}\\ f_{12d}=f_{1}-f_{2},f_{23d}=f_{2}-f_{3},f_{123d}=f_{12d}-f_{23d}\\ f_{123d}<f_{23d}<f_{12d}<f_{3}<f_{2}<f_{1} \end{array}$$

where $\varphi_{id}$ is the wrapped phase difference, $f_{id}$ is the equivalent frequency, and i=12,23,123. The phase difference operation can obtain the phases of larger equivalent periods. When selecting an appropriate combination of periods, the period of the synthetic phase $\varphi_{12}{}_{3d}$ will cover the whole measurement field, while $\Phi_{123d}=\varphi_{12}{}_{3d}$ is a continuous phase, which could help calculate the fringe orders.

The phase sum operation in the proposed method is also introduced to obtain the synthetic phases with higher equivalent frequency. The phases $\varphi_{12}{}_{s}$ and $\varphi_{23}{}_{s}$ after the first phase sum calculation are obtained by

(9) $$\{\begin{array}{c} \varphi_{12}{}_{s}=\{\begin{array}{c} \varphi_{1}+\varphi_{2},\;\varphi_{1}+\varphi_{2}\geq 0\\ \varphi_{1}+\varphi_{2}+2\pi ,\;\varphi_{1}+\varphi_{2}<0 \end{array}\\ \varphi_{23}{}_{s}=\{\begin{array}{c} \varphi_{2}+\varphi_{3},\;\varphi_{2}+\varphi_{3}\geq 0\\ \varphi_{2}+\varphi_{3}+2\pi ,\;\varphi_{2}+\varphi_{3}<0 \end{array} \end{array},$$

where subscript s refers to the phase sum operation. After the first phase sum calculation, all the phase values ranging from 0 to 2π are obtained. If these results are used directly for the second phase sum calculation, the phase sum in higher frequencies cannot be obtained correctly. Therefore, the first phase sum results need to be shifted between −π and π by

(10) $$\varphi_{is}^{\prime }=\varphi_{is}-\pi ,\;i=12,23,$$

where $\varphi_{is}^{\prime }$ is the phase sum after the shift. Then, the $\varphi_{123}{}_{s}$ is the result of the second phase sum calculation, which is expressed as

(11) $$\begin{array}{c} \varphi_{123}{}_{s}=\{\begin{array}{c} \varphi_{12s}^{\prime }+\varphi_{23s}^{\prime },\;\varphi_{12s}^{\prime }+\varphi_{23s}^{\prime }\geq 0\\ \varphi_{12s}^{\prime }+\varphi_{23s}^{\prime }+2\pi ,\;\varphi_{12s}^{\prime }+\varphi_{23s}^{\prime }<0 \end{array}\\ f_{12s}=f_{1}+f_{2},\;f_{23s}=f_{2}+f_{3},\;f_{123s}=f_{12s}+f_{23s}\\ f_{3}<f_{2}<f_{1}<f_{23s}<f_{12s}<f_{123s} \end{array}$$

where $f_{is}$ is the equivalent frequency of phase sum and i=12,23,123. The phase sum operation can obtain the phases of smaller equivalent periods.

**Figure 4:**
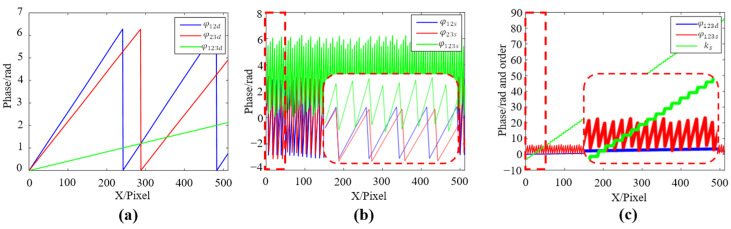
Phase calculation process. (**a**) Phase difference operation (**b**) Phase sum operation. (**c**) Phase unwrapping, $k_{s}$ is the fringe order of $\varphi_{123}{}_{s}$ (the area with a dashed line has 0 to 50 pixels).

Afterward, the phase sum $\varphi_{123}{}_{s}$ is restored to a continuous phase using phase difference $\varphi_{123}{}_{d}$. The phase calculation process is shown in Figure 4.

Figure 4a,b are the synthetic phase profiles obtained by phase difference and phase sum operations. In Figure 4c, $\varphi_{123d}$ is a continuous phase ($\Phi_{123d}=\varphi_{123d}$), while $\varphi_{123s}$ is a wrapped phase. The fringe order $k_{s}$ of $\varphi_{123s}$ and the final continuous phase $\Phi_{123s}$ can be obtained by

(12) $$\begin{array}{c} k=round\left[\frac{\Phi_{i}\left(f_{j}/f_{i}\right)-\varphi_{j}}{2\pi }\right]\\ \Phi_{j}=\varphi_{j}+2\pi k \end{array}$$

where $\Phi_{i}$ and $\Phi_{j}$ are the lower frequency and higher frequency continuous phase, $\varphi_{j}$ is the higher frequency wrapped phase (i=123d,12d,1,12s, j=12d,1,12s,123s), k is the fringe order. The sequence of the whole phase recovery is as follows: $\Phi_{123d}\to \Phi_{12d}$, $\Phi_{12d}\to \Phi_{1}$, $\Phi_{1}\to \Phi_{12s}$, $\Phi_{12s}\to \Phi_{123s}$. The phase is unwrapped from low to high equivalent frequencies step by step.

#### 2.3.3. Mathematical Derivation and Analysis

The sensitivity gain *G* (between $\Phi_{123d}$ and $\Phi_{123s}$) of the three-frequency method can be calculated according to the two-frequency method [34,35,36].

(13) $$\begin{array}{l} G=\frac{\Phi_{123s}}{\Phi_{123d}}=\frac{T_{123d}}{T_{123s}},\;T_{123d}=\frac{T_{12d}T_{23d}}{\left|T_{12d}-T_{23d}\right|},\;T_{123s}=\frac{T_{12s}T_{23s}}{T_{12s}+T_{23s}},\\ T_{12d}=\frac{T_{1}T_{2}}{\left|T_{1}-T_{2}\right|},\;T_{23d}=\frac{T_{2}T_{3}}{\left|T_{2}-T_{3}\right|},\\ T_{12s}=\frac{T_{1}T_{2}}{T_{1}+T_{2}},\;T_{23s}=\frac{T_{2}T_{3}}{T_{2}+T_{3}} \end{array}$$

Obtaining

(14) $$G=\left|\frac{T_{1}T_{2}(T_{2}+T_{3})+T_{2}T_{3}(T_{1}+T_{2})}{T_{1}T_{2}\left|T_{2}-T_{3}\right|-T_{2}T_{3}\left|T_{1}-T_{2}\right|}\right|.$$

As the $T_{1}$, $T_{2}$, and $T_{3}$ are greater than zero, the G is always greater than one. The phase sum $\Phi_{123s}$ is $G=T_{123d}/T_{123s}$ times more sensitive than $\Phi_{123d}$. Next, the SNRs of phase difference and phase sum will be discussed. In practice, the phases $\Phi_{1n}\left(x,y\right)$, $\Phi_{2n}\left(x,y\right)$, and $\Phi_{3n}\left(x,y\right)$ are corrupted by additive white Gaussian noise $n_{1}\left(x,y\right)$, $n_{2}\left(x,y\right)$, and $n_{3}\left(x,y\right)$ as

(15) $$\begin{array}{l} \Phi_{1n}(x,y)=2\pi h\left(x,y\right)/T_{1}+n_{1}(x,y)\\ \Phi_{2n}(x,y)=2\pi h\left(x,y\right)/T_{2}+n_{2}(x,y)\\ \Phi_{3n}(x,y)=2\pi h\left(x,y\right)/T_{3}+n_{3}(x,y) \end{array}$$

where $n_{1}$, $n_{2}$, and $n_{3}$ (omit the coordinate notation (x,y) henceforth) are uncorrelated samples with a variance of $\sigma^{2}$, and h(x,y) (h(x,y)=Φ(x,y)T/2π) is the height contained in the phase. The phase difference $\Phi_{123dn}$ and phase sum $\Phi_{123sn}$ with noise are

(16) $$\begin{array}{l} \Phi_{123dn}=2\pi h\left(x,y\right)/T_{123d}+\left(n_{1}-n_{2}\right)+\left(n_{3}-n_{2}\right)\\ \Phi_{123sn}=2\pi h\left(x,y\right)/T_{123s}+\left(n_{1}+n_{2}\right)+\left(n_{2}+n_{3}\right) \end{array}$$

Then, the SNRs for $\Phi_{123dn}$ and $\Phi_{123sn}$ are

(17) $$\begin{array}{l} \mathrm{SNR}(\Phi_{123dn})=\frac{{\left(\frac{2\pi }{T_{123d}}\right)}^{2}\iint_{(x,y)\in \Omega }{\left|h(x,y)\right|}^{2}d\Omega }{\iint_{(x,y)\in \Omega }{\left|n_{1}-n_{2}\right|}^{2}d\Omega +\iint_{(x,y)\in \Omega }{\left|n_{3}-n_{2}\right|}^{2}d\Omega }\\ \mathrm{SNR}(\Phi_{123sn})=\frac{{\left(\frac{2\pi }{T_{123s}}\right)}^{2}\iint_{(x,y)\in \Omega }{\left|h(x,y)\right|}^{2}d\Omega }{\iint_{(x,y)\in \Omega }{\left|n_{1}+n_{2}\right|}^{2}d\Omega +\iint_{(x,y)\in \Omega }{\left|n_{2}+n_{3}\right|}^{2}d\Omega } \end{array}$$

where the (x,y)∈Ω is the two-dimensional region where the fringe data are well-defined. Since $n_{1}$, $n_{2}$, and $n_{3}$ are generated by the same Gaussian zero-mean stationary stochastic process, then the average energies of $n_{1}-n_{2}$, $n_{1}+n_{2}$; $n_{3}-n_{2}$, and $n_{3}+n_{2}$ are equal [34,35,36,37]. The SNR gain between $\Phi_{123sn}$ and $\Phi_{123dn}$ is

(18) $$\frac{\mathrm{SNR}\left(\Phi_{123sn}\right)}{\mathrm{SNR}\left(\Phi_{123dn}\right)}={\left(\frac{T_{123d}}{T_{123s}}\right)}^{2}=G^{2}.$$

Thus, $\Phi_{123sn}(x,y)$ has $G^{2}$ times higher SNR than $\Phi_{123dn}(x,y)$. A phase sum operation can obtain higher *G* and SNR through mathematical analysis. 

In addition, the noise-induced phase error is also Gaussian distributed and is assumed to have a variance of $\sigma_{\Phi }^{2}$ according to the study of Ref [29]. After the phase unwrapping is performed, the phase is extended from 2π to $2\pi f_{t}$ ($f_{t}$ is the total number of fringes in the fringe pattern). If the already expanded continuous phase is scaled down to the scale [−π,π), the variance of the phase error is equivalent to being reduced by a factor of $f_{t}{}^{2}$.

(19) $$\sigma_{\Phi }^{2}(x,y)=\frac{2}{Nf_{t}{}^{2}}\frac{\sigma^{2}}{B^{2}}.$$

Equation (19) shows that three factors can be used to reduce the impact of noise, but the most convenient way to reduce phase error for a system that already exists is increasing $f_{t}$. Compared with the DFH method, the TFH method can select the fringes with higher frequency to calculate the phases because it performs the phase difference twice, and it can measure a larger field. This paper introduces the phase sum operation with higher sensitivity gain and SNR into the TFH method. Combining the TFH method with the phase sum operation can obtain better measurement results through mathematical derivation and theoretical analysis.

## 3. Simulation

### 3.1. Phase Calculation from Fringe Patterns with Noise Added

**Figure 5:**
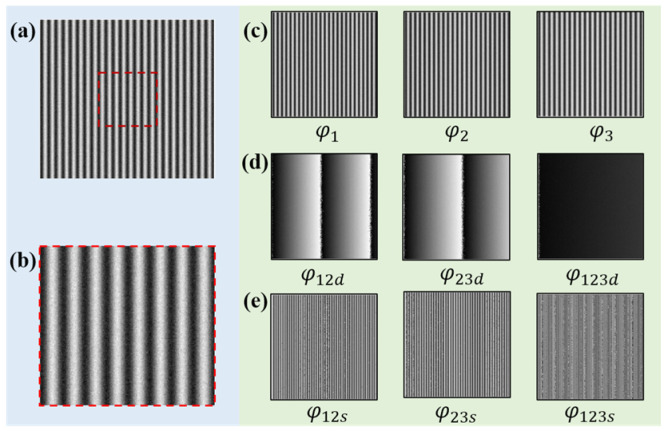
Simulation of the process from fringes to phases. (**a**) Fringe patterns with noise. (**b**) Enlarged image of the boxed area. (**c**) The phases obtained by the PSP. (**d**) Phase difference maps. (**e**) Phase sum maps.

To verify the whole process and the anti-noise performance, random noise with an SNR of 29.8 dB is added to the simulated fringes. The simulated image size is 512 × 512 pixels, and the computer generates three groups of sinusoidal fringe patterns ($T_{1}=21$, $T_{2}=23$, $T_{3}=25$). The phase is calculated by the four-step PSP algorithm. The phase calculation process with noise is shown in Figure 5.

**Figure 6:**
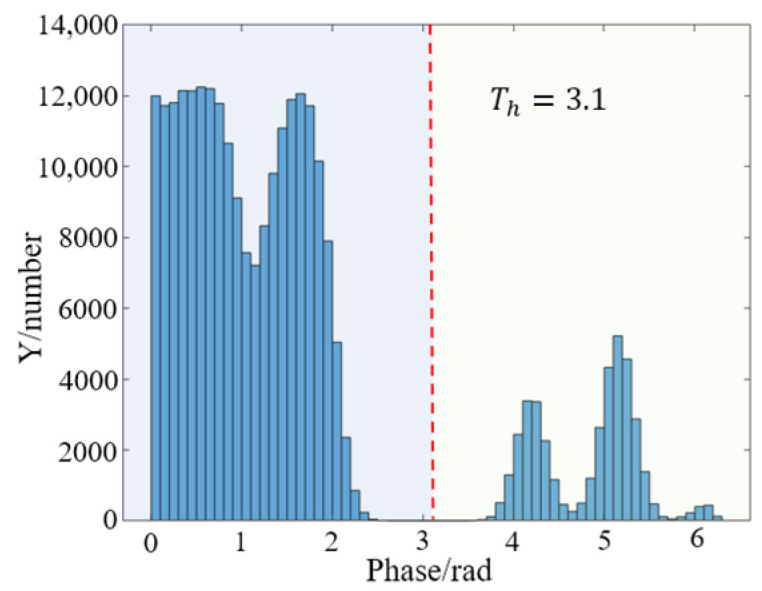
Histogram graph of $\left|\varphi_{12d}-\varphi_{23d}\right|$ with noise.

In the first phase difference calculation process, the statistics of the phase difference absolute value $\left|\varphi_{12d}-\varphi_{23d}\right|$ with noise are calculated and shown in Figure 6.

**Figure 7:**
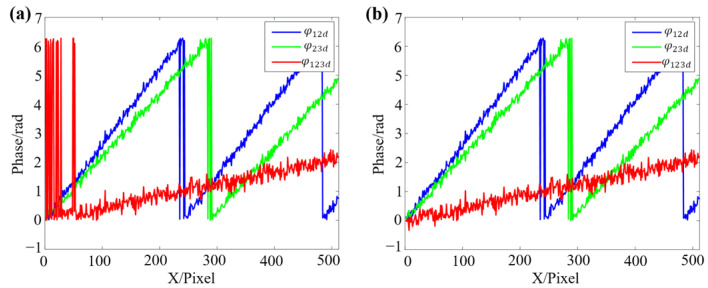
Comparison of the traditional and the proposed method in phase difference calculation. (**a**) The traditional method. (**b**) The proposed method.

It can be observed that the phase difference values after adding noise still remain in the found distribution, which could be divided into two parts. The threshold is $T_{h}=3.1$ using the Otsu method, and it will be used for the second phase difference calculation. The comparison of improved phase difference calculation results and the traditional method are shown in Figure 7. 

**Figure 8:**
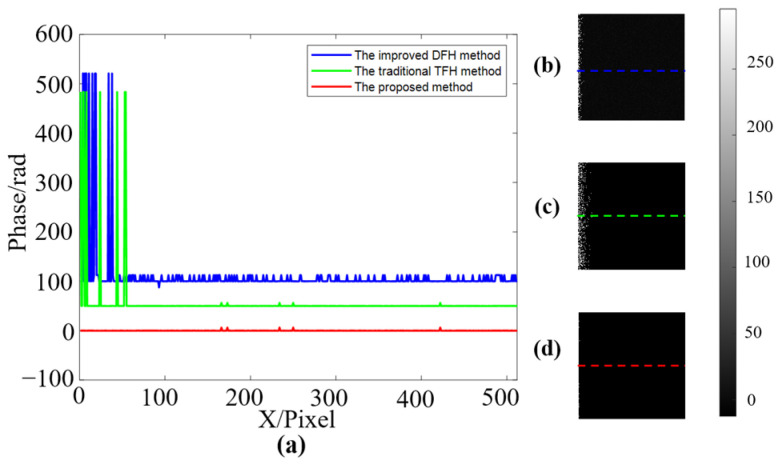
Comparison of the plane phases obtained by the three methods for a virtual plane. (**a**) Phase profiles of the three methods (each line shifts 50 rad for clarity, and the color corresponds to the right). (**b**–**d**) are the normalized plane phases recovered by the improved DFH method, the traditional TFH method, and the proposed method, respectively.

Figure 7a shows the results of traditional phase difference calculation. At the position where the phase value is close, due to the effect of noise, the calculated continuous phase has multiple jumps of 2π. Figure 7b demonstrates the result of the improved method proposed in this work, and it can effectively eliminate the phase jumps. To further study the improvement compared with other methods, one of the improved DFH methods [39], the traditional TFH method, and the proposed method are compared. To ensure that each method can finally obtain a continuous phase, the fringe periods of the improved DFH method are set as ${T^{\prime }}_{1}=31$ and ${T^{\prime }}_{2}=32$, and the fringe periods of the traditional TFH method and the proposed method are set as $T_{1}=21$, $T_{2}=23$, and $T_{3}=25$. All methods are employed to measure a virtual plane, and the same level of random noise is added to fringe patterns. The plane phase of each method is calculated and shown in Figure 8.

Comparing these three methods shows that the improved DFH method and the traditional TFH method have some steep phase jumps, and the improved DFH method has more small phase fluctuations in the linear region. In general, using the proposed method can effectively improve the accuracy of the measurement and reduce the phase jumps caused by noise.

### 3.2. Effect of Fringe Period Selection on Phase Calculation

**Figure 9:**
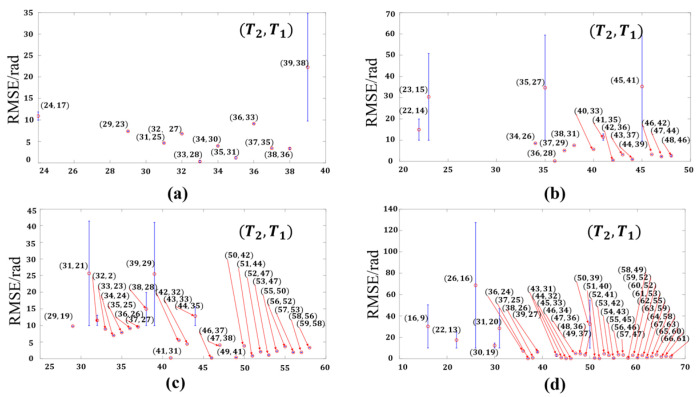
Error bar graph of $\Phi_{123s}$ for various period combinations. (**a**) $T_{3}=40$, (**b**) $T_{3}=50$, (**c**) $T_{3}=60$, (**d**) $T_{3}=70$.

Then, the selection of the fringe period is discussed and the effect of various combinations on the measurement accuracy is studied. It can be predicted that not all combinations are feasible. The combination must meet as

(20) $$\begin{array}{c} T_{1}<T_{2}<T_{3}\\ T_{12d}<m,\;T_{23d}<m,\;T_{123d}>m \end{array}$$

where m is the image resolution in the fringe coding direction. Since there are three periods that need to be determined step by step, we first fix $T_{3}$ in the range of 20 to 80, then fix $T_{2}$ in the range of 1 to $T_{3}-1$, and finally fix $T_{1}$ in the range of 1 to $T_{2}-1$. The random noise is also added to the simulation, and the root mean square error (RMSE) of different combinations is calculated after determining each period. When the maximum period $T_{3}=40,50,60,70$, we select the $T_{1}$ with the smallest error after $T_{2}$ is fixed to draw the error bar graph of $\Phi_{123s}$, which is labeled as $\left(T_{2},T_{1}\right)$. To avoid the random fluctuation caused by a single calculation as much as possible, the mean value and peak-valley value are obtained by repeating each combination 20 times. The image size is 1920 pixels × 1920 pixels. The error bar graph for various period combinations is shown in Figure 9.

**Figure 10:**
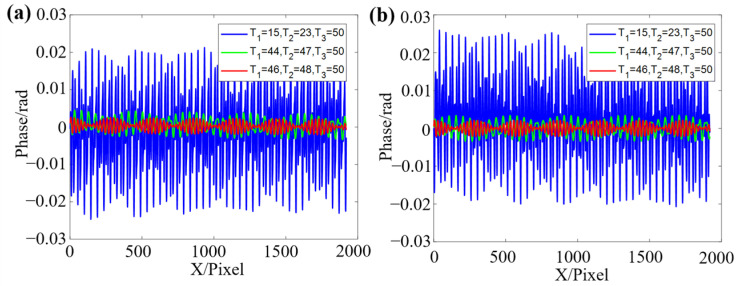
Comparison of different period combinations on phase operation results. (**a**) The error of $\Phi_{123d}$. (**b**) The error of $\Phi_{123s}$.

According to Figure 9, it is known that when the value of $T_{3}$ is larger, there are more stable period combinations that meet Equation (20). When the values of $T_{1}$, $T_{2}$, and $T_{3}$ are relatively close, the error value and fluctuation are smaller, while the error in the place where the period difference is is larger, and the fluctuation of the calculation results is also larger. Those period combinations are sensitive to noise in the measurement process. Therefore, that should be avoided. To investigate the reasons for the large errors and fluctuations generated by some combinations, the three combinations with the periods of $T_{1}=15$, $T_{2}=23$, $T_{3}=50$, $T_{1}=44$, $T_{2}=47$, $T_{3}=50$, and $T_{1}=46$, $T_{2}=48$, $T_{3}=50$ are conducted without adding noise to reflect the impact of different combinations simply. The errors of the $\Phi_{123d}$ and $\Phi_{123s}$ corresponding to the three combinations are shown in Figure 10.

According to Figure 10, it can be known that those combinations with larger RMSE in Figure 9 have a wider range of fluctuations and errors. This leads to larger phase errors and reduces the measurement accuracy during the phase calculation. Therefore, selecting the appropriate fringe period combination is necessary before conducting the experiments.

**Figure 11:**
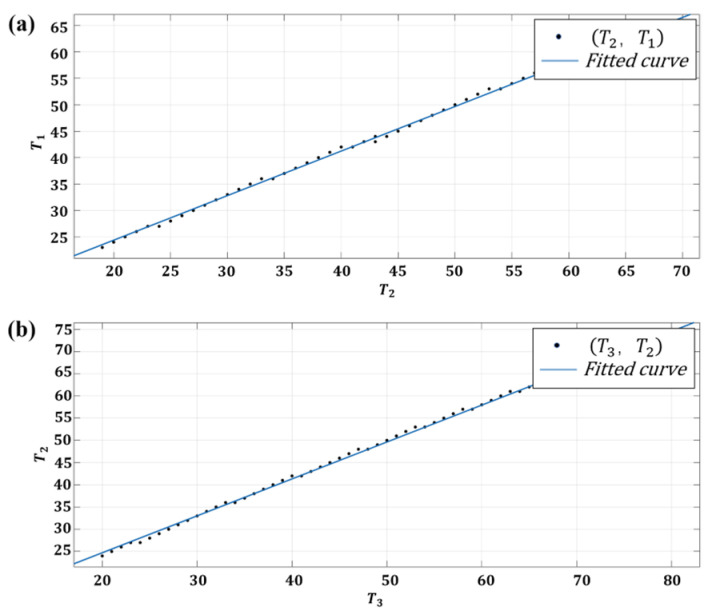
Optimal combination between $T_{1}$ and $T_{2}$ and $T_{2}$ and $T_{3}$. (**a**) Curve fitting between $T_{1}$ and $T_{2}$. (**b**) Curve fitting between $T_{2}$ and $T_{3}$.

To obtain the optimal period combination, those points with lower mean and peak-valley values are selected, as shown in Figure 11. It can be seen that the optimal combination of periods shows a linear trend. Therefore, it can be fitted by a linear function, Equation (21).

(21) $$\begin{array}{l} T_{2}=round\left(k_{1}T_{3}+k_{2}\right)\\ T_{1}=round\left(k_{3}T_{2}+k_{4}\right) \end{array}$$

Using the fitting function, the values of $k_{1}$, $k_{2}$, $k_{3}$, and $k_{4}$ can be obtained, respectively, as

(22) $$\begin{array}{l} k_{1}=0.9691\\ k_{2}=-0.2224\\ k_{3}=0.9340\\ k_{4}=1.4980 \end{array}$$

Before the fringe projection, the maximum period needs to be first determined for the measurement, and then the periods of the other two groups are obtained by function calculation. The accuracy of the measurement results can be further improved through this process.

## 4. Experiment

**Figure 12:**
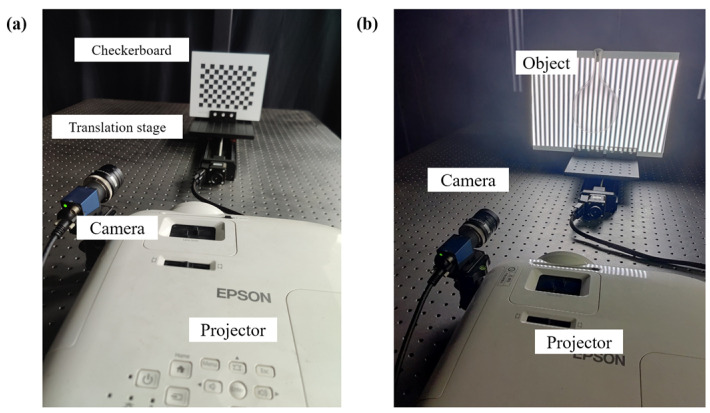
Experimental measurement system. (**a**) Calibration scheme. (**b**) Measurement scheme.

A fringe projection measurement system is set up for the experiment, as shown in Figure 12. The system includes a CCD camera, a digital projector, a high-precision motorized linear translation stage, a checkerboard, and a computer. The CCD camera is the digital camera IMAVISION MER-231-41GM-P of the Mercury series from Daheng Imaging, with a resolution of 1920×1200. The motorized linear translation stage is a GCD-203300M from Daheng Optics, with an accuracy of 0.001 mm. The projector is an Epson CH-TW5600, with a resolution of 1920×1080. The size of the checkerboard square is 15 mm.

**Figure 13:**
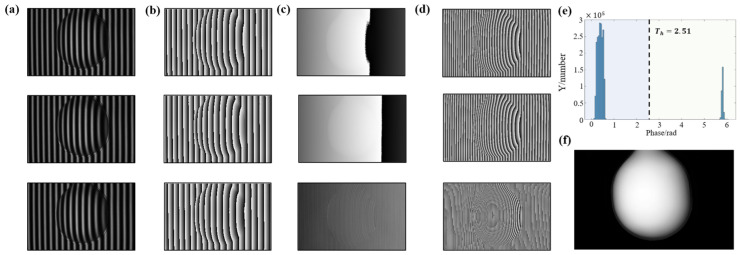
The calculation process of the proposed algorithm on an actual object. (**a**) Deformed fringe patterns. (**b)** Wrapped phases obtained by PSP. (**c**) Phase difference maps. (**d**) Phase sum maps. (**e**) Histogram graph of $\left|\varphi_{12d}-\varphi_{23d}\right|$. (**f**) Final continuous phase $\Phi_{123s}$.

Under the premise of satisfying the period relationship of Equation (20), the maximum period is selected to be $T_{3}=60$, so that the continuous phases can be obtained for fair and reliable comparison when other methods are calculated, which will be discussed later. According to the period optimization method, the other two groups of fringe periods are $T_{2}=58$ and $T_{1}=56$. Firstly, the smooth surface continuous objects are measured, as shown in Figure 13.

Figure 13a shows the deformed fringe patterns, and Figure 13b illustrates the wrapped phases calculated by PSP. Figure 13c describes the phase difference maps. After two phase difference operations, the continuous phase covering the whole measurement field is obtained. Figure 13d shows the phase sum maps, and the equivalent frequency after two phase sum operations is much more than that of all projected fringes. Figure 13e demonstrates the histogram graph of $\left|\varphi_{12d}-\varphi_{23d}\right|$, and it still conforms to the found distribution. The threshold obtained by the Otsu segmentation algorithm is 2.51. Figure 13f illustrates the final continuous phase $\Phi_{123s}$, obtained by the introduced phase recovery sequence.

The calibration is performed using a translation stage and a checkerboard to present the results in the world coordinate system. The translation stage moves with a known height multiple times to calculate the phase-to-height parameter [40]. The calibrated height volume is 100 mm. Twenty-five checkerboard images are captured and calculated for camera internal, external, and distortion parameters by Zhang’s camera calibration technique [41]. After calibration, the subsequent experimental results are converted to the world coordinate system.

To make the error comparison fairer and more reliable, a 24-step phase-shifting plus multi-frequency method [28] is used to obtain the ground truth in the experiment. The periods of the projected fringe are determined by ${T_{i}}^{\prime \prime }=\frac{m}{2^{i-1}}$ (i=1,2,3,…), where the projector’s resolution is m×n, and m and n are the horizontal and vertical resolutions of the projector, respectively.

Then, the comparison of the proposed method, the traditional TFH method, and the improved DFH method is performed. To ensure that these three methods can obtain continuous phases, and make their period values close to proceed with a fairer comparison, the periods of DFH are selected as $T_{2}{}^{\prime }=68$ and $T_{1}{}^{\prime }=66$, while the combination of the proposed method and the traditional TFH method are selected as $T_{3}=60$, $T_{2}=58$, and $T_{1}=56$. The target of this combination with a smaller period value is to avoid $f_{id}>m$ (i=12,23) after the first phase difference calculation, where m is the horizontal resolution of the projector (m=1920 in this research). Additionally, the periods of the multi-frequency method are determined by ${T_{i}}^{\prime \prime }=\frac{1920}{2^{i-1}}$ (i=1,2,3,4,5,6), and the continuous phase is solved with ${T_{6}}^{\prime \prime }=60$ as the ground truth. In this case, each method can satisfy its own period relationship.

**Figure 14:**
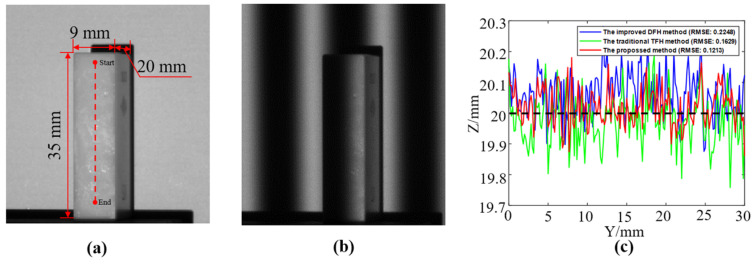
Measurement on a ceramic standard gauge block. (**a**) Dimension of the gauge block (the red dotted line refers to the area where the three methods are compared). (**b**) One of the captured deformed fringe patterns. (**c**) Comparison of the results obtained by the three methods along the red dotted line (the RMSE of each method is marked).

A ceramic standard gauge block with a height of 20 mm was measured. A linear area was selected on its upper surface to compare these three methods, as shown in Figure 14.

**Figure 15:**
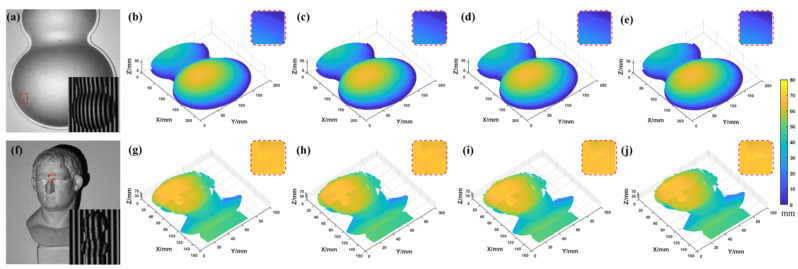
Measurement of a gourd and a statue. (**a**,**f**) The gourd and statue with their deformed fringe pattern. (**b**,**g**) The ground truth. (**c**,**h**) The results of the proposed method. (**d**,**i**) The results of the traditional TFH method. (**e**,**j**) The results of the improved DFH method (the small images in the upper right are enlarged images of the selected region).

As shown in Figure 14, the result of the proposed method is closer to the actual height, and the RMSE is smaller. A part of a gourd and a statue were measured to compare the three methods further. The comparison between these methods was performed, as shown in Figure 15.

**Figure 16:**
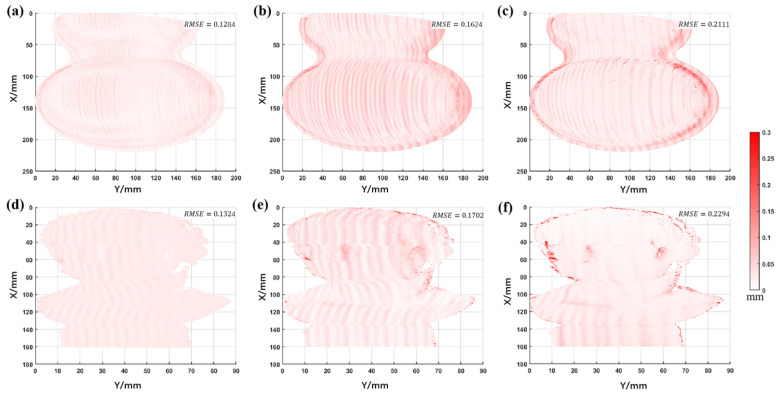
The ED maps between the three methods and the ground truth (the first and the second rows refer to the measured gourd and statue, respectively). (**a**,**d**) The ED map of the proposed method. (**b**,**e**) The ED map of the traditional TFH method. (**c**,**f**) The ED map of the DFH method (the RMSE of each method is marked).

Analyzing the results in Figure 15, the proposed method has fewer jump points than the other two methods. To show more clearly the differences between several methods, their Euclidean distance ($ED=\sqrt{{\left(x-x^{\prime }\right)}^{2}+{\left(y-y^{\prime }\right)}^{2}+{\left(z-z^{\prime }\right)}^{2}}$, where (x,y,z) and $\left(x^{\prime },y^{\prime },z^{\prime }\right)$ are the coordinates of two space points, respectively) maps were calculated from the ground truth, as shown in Figure 16.

Figure 16 shows that the overall RMSE of the proposed method is minimal, and there are fewer jump points. The above comparison proves that the proposed method can further improve measurement accuracy and reduce jump errors.

**Figure 17:**
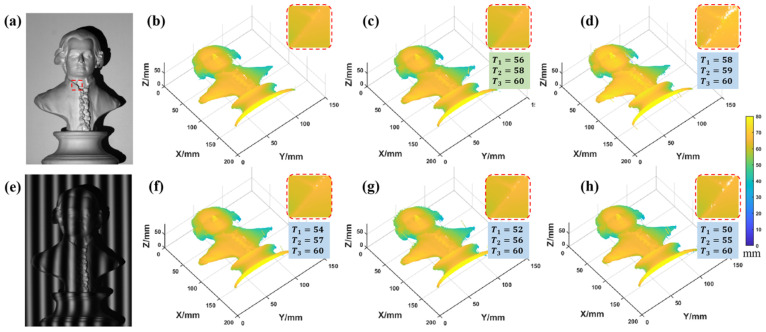
Measurement results under different period combinations. (**a**,**e**) The statue and deformed fringe pattern. (**b**) Ground truth. (**c**) Combination 1: 56, 58, 60 (combination 1 is optimal combination). (**d**) Combination 2: 58, 59, 60. (**f**) Combination 3: 54, 57, 60. (**g**) Combination 4: 52, 56, 60. (**h**) Combination 5: 50, 55, 60 (the small images in the upper right are enlarged images of the selected region).

The optimization method of the fringe period is verified. According to the above conclusion, the optimal fringe periods are $T_{1}=56,\;T_{2}=58,\;T_{3}=60$, and the results are compared with several other periods’ results, as shown in Figure 17.

**Figure 18:**
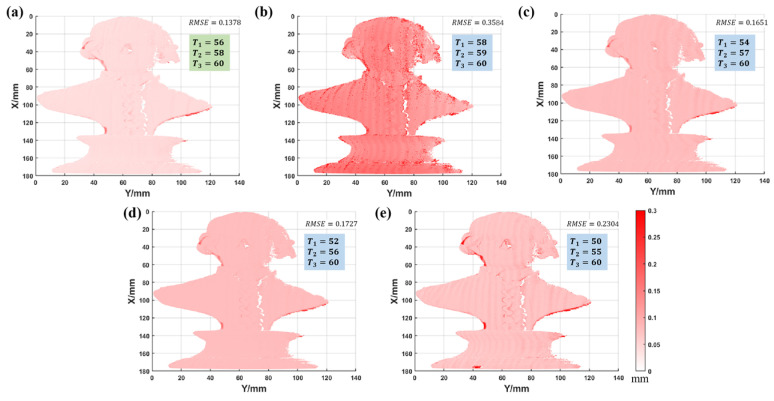
The ED maps between the different period combinations and the ground truth. (**a**) Combination 1: 56, 58, 60 (combination 1 is optimal combination). (**b**) Combination 2: 58, 59, 60. (**c**) Combination 3: 54, 57, 60. (**d**) Combination 4: 52, 56, 60. (**e**) Combination 5: 50, 55, 60 (the RMSE of each combination is marked).

The optimal combination has much fewer jumps and is almost the same as the ground truth. The error of other combinations is more prominent, especially combination 2. To more clearly observe the difference between the results of several combinations and the ground truth, the ED maps were calculated as shown in Figure 18.

As shown in Figure 18, the ED map and RMSE of the optimal combination are the smallest, while the error of combination 2 is the largest, and the error level of combination 3, 4, and 5 is between them. Figure 18 shows that the optimal combination has the least phase jumps and a higher measurement accuracy. This experiment proves that the optimal period selection criterion can obtain better measurement results.

The above experiments can show that the proposed method is feasible and has a high anti-noise ability. By comparing the results obtained by several methods, it can be seen that the proposed method has better measurement accuracy, also with the optimal fringe period combination.

## 5. Conclusions

An improved TFH synthesis phase unwrapping algorithm is proposed in this research. The phase sum operation is introduced into the TFH algorithm, and the phase sum and phase difference operations are improved. In the process of phase difference calculation, according to the found distribution of phase difference value, the Otsu method is used to calculate the threshold to help phase judgment and reduce the effect of noise. In the process of phase sum operation, the phase with a much higher frequency than those of projected fringes can be realized. The continuous phase difference is used to assist in phase sum unwrapping. The improved phase sum and phase difference operation jointly achieve the effect of accuracy improvement and error reduction. The fringe period selection method is deduced based on the distribution of error values for different period combinations, and better results can be obtained using the optimal combination. Simulation and experimental results show the feasibility and anti-noise ability of the proposed method, which can improve the accuracy of measurement results to a certain extent.

## Data Availability

Not applicable.
